# Overcoming Dormancy in *Prunus* Species under Conditions of Insufficient Winter Chilling in Israel

**DOI:** 10.3390/plants13060764

**Published:** 2024-03-08

**Authors:** Amnon Erez

**Affiliations:** The Volcani Center, Institute of Plant Sciences, P.O. Box 15159, Rishon LeZion 7505101, Israel; erezamn@gmail.com

**Keywords:** stone fruits, chilling requirements, chilling portions, bud break, dynamic model, dormancy avoidance

## Abstract

The phenomenon of dormancy and the evolutionary causes for its development are presented together with the effects of the climatic factors: temperature and light. Shade and darkness have been found to enhance bud breaking in peach. The effects of various temperatures on chilling accumulation, chilling negation and chilling enhancement are described. The way these are computed in the face of global warming is explained, using the dynamic model. When natural chilling is less than that required, there are ways of compensation, up to a certain level. Various horticultural, physical and chemical means to achieve this are described, including bending branches, reducing vegetative vigor, shading the orchard, sprinkling to reduce daytime temperature and the application of various chemicals to break dormancy. When winter chilling is markedly reduced and temperatures increase considerably, the use of dormancy avoidance is suggested in frost-free places. This technique can induce a new growing cycle by avoiding dormancy altogether. However, the best approach is to breed high-quality cultivars requiring much less chilling. Another aspect discussed in this work, independent of the chilling requirement, is the negative effect of heat spells in winter and spring on the abnormal development of flower buds, leading to a low level of the stone fruit set and a reduced yield.

## 1. Introduction

This paper is a summary of relevant ideas and concepts developed during my activity coping with the adaptation of various species of the *Prunus* genus to the warm weather in Israel.

Even before the establishment of Israel in 1948, the interest in deciduous fruit species known to the immigrants coming from Europe led to trials to cultivate them in this different climate. Research in this area, initiated by Samish [1] and continued by myself and others [2,3,4,5,6,7,8], resulted in a system by which this became possible. In this paper, I describe the means developed over a long period of time, with the aim of helping countries that, because of global warming, are now exposed to warmer climates than previously.

The major problem for perennial plants growing in a temperate climate is how to cope with winter chilling. Contrary to animals, plants cannot change their location and need to find another solution. Deciduous fruit trees found the way through evolution: they drop susceptible parts—their leaves—and develop cold resistance in the remaining organs.

Three main problems had to be solved in these trees: 1. How to predict the approaching winter and to prepare for it. 2. How to survive the cold period. 3. How to “know” that winter, with its risk of cold, is over and that growth resumption is safe. The environmental factors perceived by the plant, indicating the approach of the cold season, are the decreasing daylength and the fall in temperatures. These factors induce four responses in the deciduous tree: (i) the cessation of terminal growth, (ii) leaf drop, (iii) development of cold resistance and (iv) the induction of dormancy in the buds. This strategy allows the plant to survive low winter temperatures. How will the buds “know” when to renew growth with no risk of damage? For this purpose, dormancy was developed.

Dormancy is defined as the sum of processes that create a situation wherein embryonic tissues are unable to grow, even under environmental conditions that generally favor growth and development [9]. Only embryonic tissues above the ground go into dormancy, i.e., floral and vegetative buds, and the cambium. The dormancy of the latter is dependent on bud dormancy. There is no deep dormancy in the roots. The dormant organs perceive and react to environmental information. The dormant period is divided into three parts according to Lang et al. [9]: (i) Paradormancy is where the lateral buds are correlatively inhibited by the terminal buds. In this period, re-growth is possible by cutting the apex. (ii) Endodormancy is the true dormancy that is overcome naturally by cold. (iii) Ecodormancy is where growth is limited by unsuitable environmental conditions, mostly excessively cold temperatures. Faust et al. [10] divided endodormancy further into two periods: D-endodormancy, where dormancy is deep, and S-dormancy, a shallower dormancy where the buds may react to dormancy-breaking agents and start growing.

The dilemma in “deciding” when to renew growth is that while the dormant buds can withstand very low temperatures, once growth starts, cold resistance disappears. As growth resumption is a one-way track, renewed cold exposure following growth resumption may lead to the death of the entire tree. To avoid this, the mechanism of dormancy was developed by evolution. Dormancy requires a specific exposure to chilling before the buds can resume growth. This may ensure that growth will start only when the chilling requirement is met. Each cultivar has its own chilling requirement adapted to the location where it developed. “Chilling” is a specific level of temperatures’ time duration as described below. Every bud responds individually to the conditions to which it is exposed. During dormancy, the connection between various plant organs is severed as plasmodesmata become blocked [11].

## 2. Temperatures Effect on Dormancy

**Figure 1:**
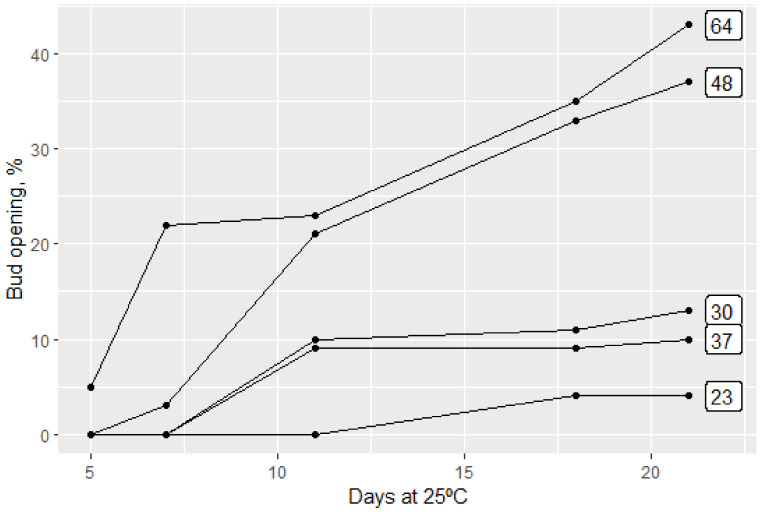
Breaking of vegetative buds of *Bonita* peach during forcing after different durations of exposure to days (squared on the right side) at 4 °C. (Buds on excised branches exposed to continuous chilling at 4 °C in the dark. Forcing at 25 °C in the light, bud break in %).

The three effects of efficient dormancy breaking are a high level, uniformity and precocity of bud breaks, i.e., the need of heat accumulation for bud opening is low. This is shown in Figure 1.

A trial of exposing cut peach branches (12 per treatment), taken in autumn 1971, to increasing periods of chilling at 4 °C followed by forcing at 25 °C is shown.

We have examined the response of dormant small peach trees to different temperatures under controlled temperature conditions [12,13,14,15]. On the basis of these studies, the effects of temperature can be summarized as follows:

- Effective temperatures to break dormancy in the peach are between −2 °C and 13 °C, and the most effective being 4–8 °C with reduced efficiency at higher and lower temperatures.
- Moderate temperatures between 13 °C and 16 °C that will not break dormancy alone, when occurring in a daily cycle after previous chilling, enhance the effect of chilling. On the other hand, temperatures higher than 18 °C in a daily cycle will nullify former chilling.
- This negative effect of high temperatures increases the longer the duration and the higher the temperature. However, when cycles are longer than a day, the chilling effect is final and cannot be nullified by high temperatures.

**Figure 2:**
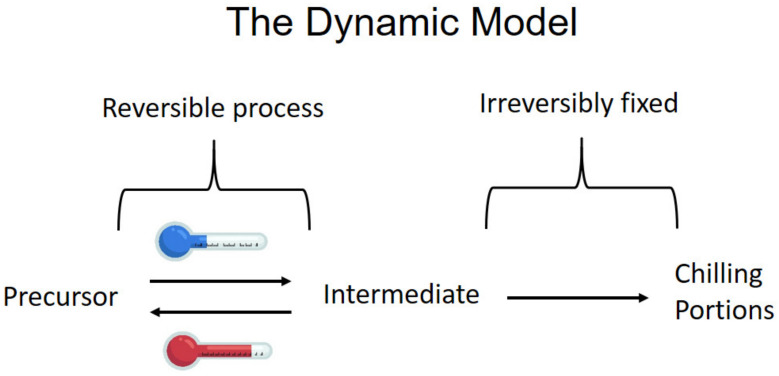
Scheme of the dynamic model.

Several models were suggested to quantify the amount of chilling based on measuring air temperatures. The most common are the chilling hours model [16], the Utah model that measures chilling units [17] and the dynamic model measuring chilling portions that was developed based on the results and concepts presented above [18,19]. Figure 2 presents a scheme of the dynamic model. The dynamic model explains and summarizes all the effects of temperatures on dormancy. It is based on two reactions in the dormant buds: The first creates a temporary product (named “intermediate” in Figure 2) due to the chilling effect; this reaction can be negated by high temperatures that will decrease the temporary product. The second reaction changes the temporary product to the final one. For the temporary product to transform to the final one, it has to reach a specific critical level. Once this level has been attained and the required portion of the final product has been produced, the temporary product level diminishes to zero, but starts accumulating again with further exposure to chilling. As a guideline, one chilling portion is obtained after exposure to 6 °C for about 28 h. With different temperature combinations, this period will be different. These portions were named “chilling portions” and represent the accumulated chill to be compared with the chilling requirements, which are specific to each cultivar, to elucidate the moment of the endodormancy release.

When running the model with meteorological temperature data, one has to take into consideration that the provided temperatures are measured in the shade. On bright days, direct solar irradiation increases bud and bark temperatures above that in the shade. This difference appears to increase with the rise in day temperature. Differences of 3–5 °C have been recorded [20]. With global warming, this difference increases, leading to greater discrepancy. Thus, a correction of the measurements is required. Using thermistor needles inserted in representative buds or exposed but covered with material having a similar response to that of buds will give more reliable data. This model has been accepted as the best so far to describe the chilling requirements of *Prunus* dormant buds [21,22].

## 3. Light Effects on Dormancy

Apart from the effects of temperature on dormancy, we also detected light effects in peach trials [23,24]. Clearly, the dormant vegetative buds perceive light signals and react to light during dormancy and bud breaks. On the other hand, flower buds in peach were found to be non-responsive to light [23]. Dormancy in vegetative buds is induced by short days. During endodormancy, the limitation of light and even total darkness enhance bud breaks in spring, compared to buds receiving natural light. But darkness in spring will prevent vegetative bud breaks even following sufficient chilling during winter [24]. So, there is an analogy between chilling and darkness.

## 4. Action on the Orchard Level to Improve Bud Breaks under Conditions of Lack of Winter Chilling

Clearly, the direct (but also the slowest) way to improve bud break under insufficient chilling conditions is to breed low-chilling-requiring cultivars. We do have a large genetic pool that can serve this purpose [25]. This work is being carried out in temperate and subtropical parts of the world where *Prunus* species are grown and climate change is strongly affecting the winter chilling accumulation. This approach will not be discussed here.

With global warming, the risk of insufficient winter chilling is threatening locations that were not aware of this problem in the past [22,26,27]. It is therefore relevant to explore the means we have to combat this situation. We can reduce the effect of climatic change and improve bud breaking by four ways [26]: (i) horticultural means, (ii) physical means, (iii) chemical means, (iv) dormancy avoidance.

### 4.1. Horticultural Means

We have two horticultural means to improve bud break in deciduous fruit trees [26]:

The first is by bending branches horizontally. Different buds have different chilling requirements; the terminals require less chilling than the laterals to overcome dormancy. Hence, when partial chilling is experienced, in many cases, the first to break will be the terminal buds, especially in upright growing species like cherry and plum, while the laterals will still be dormant. This will create a situation of apical dominance whereby the lateral buds that could otherwise break later will not break. Changing branch orientation to the horizontal position will allow the laterals to break. This is especially important with young trees.

The second means is by reducing vegetative vigor. Despite the genetic component of the chilling requirement, it may be strongly modulated by growth vigor. The higher the growth vigor of branches, the higher will be the chilling requirement of the buds on them. Reducing vigor is achieved by decreasing N nutrition, controlling irrigation, using dwarfing rootstocks or applying growth retardants.

### 4.2. Physical Means

Two means have been found to be effective in reducing chilling requirements or in helping to achieve them: shading the orchard and evaporative cooling during the warm period of the day. Shading by itself was found to have a very positive effect on dormant buds by reducing their chilling requirements [23,24]. In addition, on bright days when temperatures are high, shading prevents the increase in bud temperature above that of their environment. Covering orchards with dark nets will do just that but will also interfere with the temperature drop at night, thus reducing chilling accumulation. A solution to this problem was suggested by using vertical shade that could cast shade on the adjacent row without affecting buds’ temperature at night.

**Figure 3:**
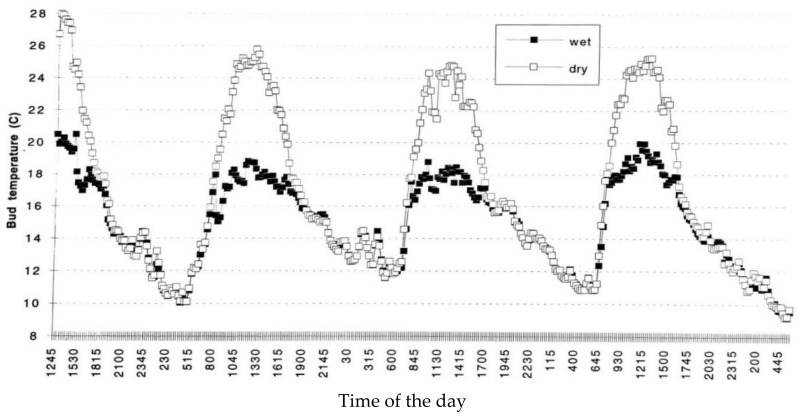
The effect of overhead sprinkling on temperature of wet buds (closed circles) compared to dry buds (open circles). The system operated on–off above 18 °C. Bud temperature was measured using tiny thermocouples. Wetting the tree lessened bud temperature markedly during the day and based on that, high negating temperatures were avoided.

Another physical means to improve bud breaks following insufficient winter chilling is the use of evaporative cooling to lessen high daytime temperatures. This is especially important when day temperatures rise above 18 °C and may nullify previous night chilling. One of the dangers of global warming is the rise in maximum temperatures. Using intermittent water sprinkling on the tree canopy at temperatures above 18 °C will decrease bud temperature considerably (Figure 3) [20]. The trial was set in a nectarine orchard in Bet Dagan, Israel, in 1982 during a warm period in winter.

### 4.3. Chemical Means

Israel was a pioneering state in the use of chemicals to break dormancy. Trials and commercial use began, even before Israel was declared a state in 1948, by Samish [1,28]. The combination of dinitro cresol and mineral oil to break dormancy of all deciduous fruit trees has been in use since 1945. This practice enabled the introduction of many species and cultivars requiring more chilling that was experienced in various locations of the country. The pioneering work of Samish was followed by Erez and coworkers, who established a wide system of chemical means to break dormancy [3,4,5,6,29]. As many of the chemicals used originally were found with time to be toxic to humans, new and other safer chemicals were developed [26]. This work is still in progress by Crane et al. [8].

The first material to gain wide acceptance as a dormancy-breaking agent was the combination of mineral oil and DNOC. When DNOC was found to be toxic to humans, other dinitro compounds were examined. Methyl dinocap successfully replaced DNOC [7] and is now in use in Israel and South Africa. The effect of the dinitro compound, being an uncoupler of oxidation and phosphorylation, is to enhance respiration. In combination with oil sprays, this spray leads to the depletion of oxygen from the oil-covered tree, causing temporary anaerobiosis. The products of anaerobic respiration, alcohol and acetaldehyde, lead to bud breaks. This treatment is very sensitive to the temperature after spraying, strongly affecting respiration. For the best effect, high temperatures following treatment are required [30].

Another important chemical is hydrogen cyanamide or Dormex^®^. This chemical was first found as active on grapes by Shulman and co-workers [31] and was later examined for many deciduous fruit trees and found to have strong activity, mostly on vegetative buds [29]. Human toxicity led to searching for replacements by other chemicals.

Other chemicals having a dormancy-breaking effect are thiourea and KNO_3_. Thiourea was very effective but was later found to have human toxicity and is not available for commercial use. KNO_3_ had a rather mild effect mostly on flower buds [29]. It was found that the reason for this was its difficulty to penetrate the buds. For that purpose, Armobreak^®^, a chemical having the property of penetration into the scale-covered bud, was found to markedly enhance the effect of added chemicals. This product was originally marketed by Akzo Nobel. This chemical, defined as a fatty amine, was found to enhance the activity of KNO_3_, gibberellic acid and other active chemicals. A strong effect was obtained especially with nitrates [6].

Of the plant hormones, two were found to have dormancy-breaking effects: gibberellins and cytokinins. Gibberellic acid may enhance especially vegetative buds. The cytokinin thidiazuron (TDZ) was found to act as a strong dormancy-breaking agent. The two chemicals in commercial use today in Israel are nitrates with Armobreak^®^ and thidiazuron, alone or with mineral oil. Commercial products of these two chemicals are available in Israel: the first under the name “Armo N” and the second under the name of “Pickup”.

**Figure 4:**
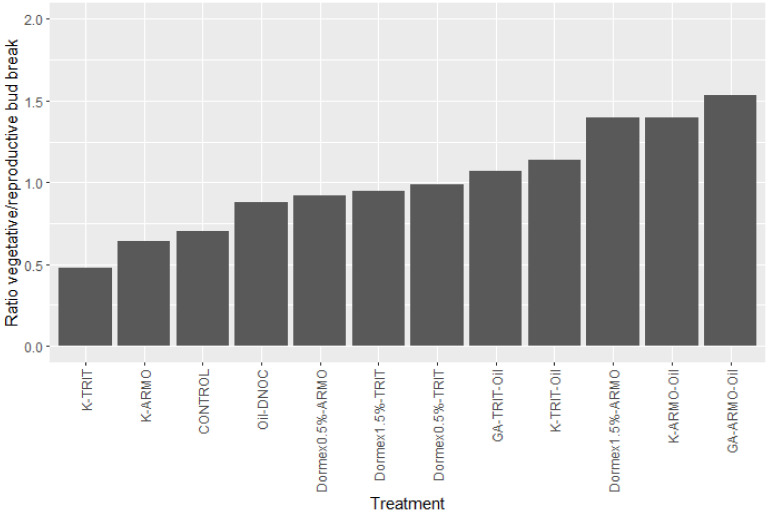
Effect of spray with different dormancy-breaking chemicals on the relative advancement in vegetative and reproductive buds in *Rhodes* peach in the coastal plain of Israel in 1995 (K = 5% potassium nitrate; Armo = 1% armobreak; Oil–DNOC = 5% oil + 0.12% DNOC; Dorm = Dormex at different concentrations in %; Trit = Triton × 100 spreader at 0.025%; Oil = 5% oil; GA = gibberellic acid at 50 ppm) [32].

The timing for the application of dormancy-breaking chemicals is when at least 2/3 of the required chilling of the cultivar has been achieved. Most of the chemicals may harm the developing bud and should be applied prior to bud swelling. The flower bud is the most sensitive organ of the tree. It should be mentioned that buds may accumulate chilling also after chemical treatment. The various chemicals used for breaking dormancy differ widely in their effect on the bud type in stone fruit species. Most of them have a much stronger effect on the break of vegetative buds than of floral buds (Figure 4). Potassium nitrate is the only chemical that has a stronger effect on floral buds. Advancement in vegetative growth may reduce the fruit set [26].

The advantages of using chemicals to break dormancy are a uniform bud opening, and an early bud break, especially if the agent is applied early (Table 1). This advancement in the bloom is important for two purposes: to obtain an earlier harvest and also to achieve a better bloom synchrony in cross pollination, especially if the two cultivars differ in their chilling requirement. Spraying the late bloomer can advance its bloom to overlap with the other cultivar. Still, it should be said that at the utmost level, chemicals can compensate for up to a third of the chilling requirement.

Clearly oil–thidiazuron at 75 and 100 ppm had a much stronger effect on both flower and leaf bud breaks, as well as on the early harvested fruit [7].

**Figure 5:**
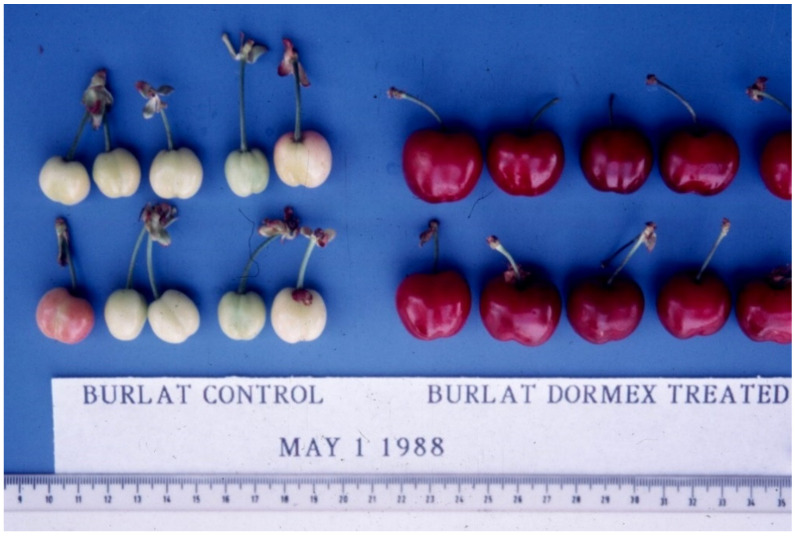
*Burlat* cherry trees treated on 21 February 1988 with 3% Dormex (**right**) and control (**left**). Picture taken on 1 May 1988 in Israel’s Southern Hills.

A demonstration of the effect of Dormex to enhance the bloom and hence to advance the harvest is shown in Figure 5. The harvest was markedly advanced by Dormex as compared to the untreated control.

### 4.4. Dormancy Avoidance

Under conditions where winter temperatures are expected to rise considerably and chilling deficiency will be very severe, we may face a situation where none of the tools that we have will compensate sufficiently for the lack of chilling. Under such conditions, we still have one remaining tool, namely dormancy avoidance. This technique has been used for a long time in the tropics where breaking dormancy by chilling is not feasible either because of a total lack of chilling or, at high elevations, due to high daytime temperatures that negate former night chilling. The dormancy avoidance technique is based on inducing the trees to initiate a new growth and bloom cycle during paradormancy, prior to the onset of endodormancy. This technique works without connection to the actual chilling requirement of the cultivar and is in use with various species of deciduous fruit trees [31]. This technique, which induces an early start of growth, leads in the tropics to shortening the growing cycle to less than one year, thus obtaining more than one crop a year. This is possible only where no temperature seasonality exists and the change in photoperiod is minimal. The cycle is reduced from 12 months to an 8- or even 6-month cycle. Thus, the year-round production of a specific fruit is possible by having plots that initiate the cycle at different times. In the tropics, the major means for enhancing bud breaks is by defoliation, mostly manually [31]. This technique may be markedly improved by the application of other means, as mentioned above. Also, as the chilling requirement is irrelevant, various high-quality cultivars can be grown by this method.

**Figure 6:**
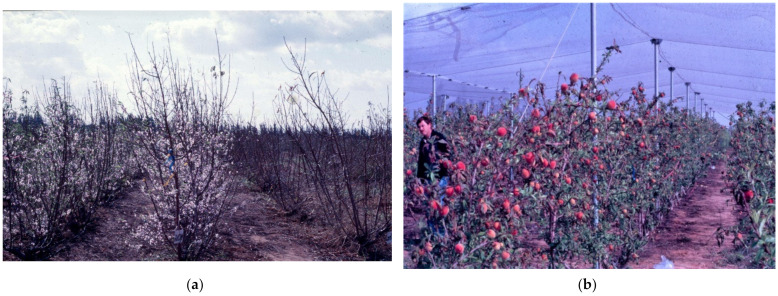
(**a**): The two left rows of *Maravilla* peach were treated in early-November 1989 and the right row was left as untreated control; picture taken in Bet Dagan on 1 December 1989. (**b**) *Earligrande* peach, in Bet Dagan, in early-March 1990 [34,35].

**Figure 7:**
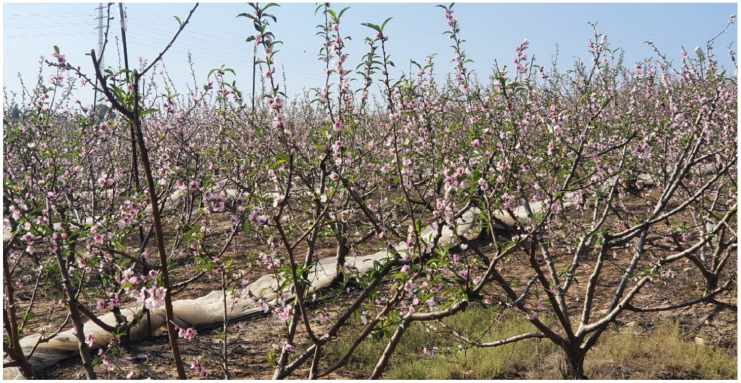
Closeup on bud break in the peach *Oded* on 20 December 2022 after chemical defoliation and spray with oil methyl dinocap in mid-November in a commercial orchard in southern Israel.

We have examined this system in the subtropical climate of Israel [23,32,33]. It can only work when the winters are mild and no frost or snow will occur in winter, and when the trees will be protected against hail by overhead nets. We used a combination of three elements to achieve good bud breaks: desiccation followed by irrigation; chemical defoliation; and later a chemical dormancy-breaking agent. The timing is critical as the vegetative buds of stone fruit species respond strongly to daylength. They enter endodormancy when daylength reaches 10 h at the end of November, irrespective of temperature. Flower buds, on the other hand, respond mostly to low temperatures when entering the endodormant state. Only when a cold spell occurs will flower buds enter endodormancy. With warming winters, such a spell may occur in Israel only in December or even January. We found that as of early November, when daylength is 10.5 h, vegetative buds are still responsive and good bud breaks can be obtained with both bud types, as a cold spell is very rare that early (Figure 6 and Figure 7). The short days following bud break induce a secondary dormancy to the growing apex, after producing just a few leaves. The flower buds develop normally, although the pollen does not separate into single pollen grains. Nevertheless, there is no shortage of normal developing fruitlets. The limiting factor in this system under conditions in Israel is the low area of photosynthesizing foliage. This can be overcome by spraying gibberellic acid at 100 ppm, which leads to renewed growth of the terminal bud when the weather is warm. Cold spells induce deeper dormancy and delay this effect. A new wave of vegetative growth will appear in February following winter chilling in peaches requiring low chilling.

The advantage of this technique is the possibility of producing fruit without chilling and that the harvest is much earlier than normal (Figure 6b). Still, it works best with low-chilling cultivars as the renewed vegetative growth depends on the chilling obtained later. Quality of the fruit is not always satisfactory, as a result of a low level of carbohydrates due to low foliage cover.

## 5. Specific Heat Effect on Stone Fruit Flower Buds

Flower buds of stone fruit species are very sensitive to high winter temperatures. After partial chilling, a heat spell may negatively affect the development of the flower parts, especially the style. The problem with this is that the damage cannot be avoided [36]. We do not know precisely the high temperature level and duration that may cause this. From trials under controlled conditions, it was found that temperatures above 30 °C are extremely injurious. This effect is independent of the chilling that accumulates later and has not been found in pome fruit species. The patterns of damage to the flowers are development of double-style fruits, short styles or complete lack of styles. This phenomenon should be further examined to specify the precise temperature effects as well as the relative sensitivity of the various species and cultivars.

**Figure 8:**
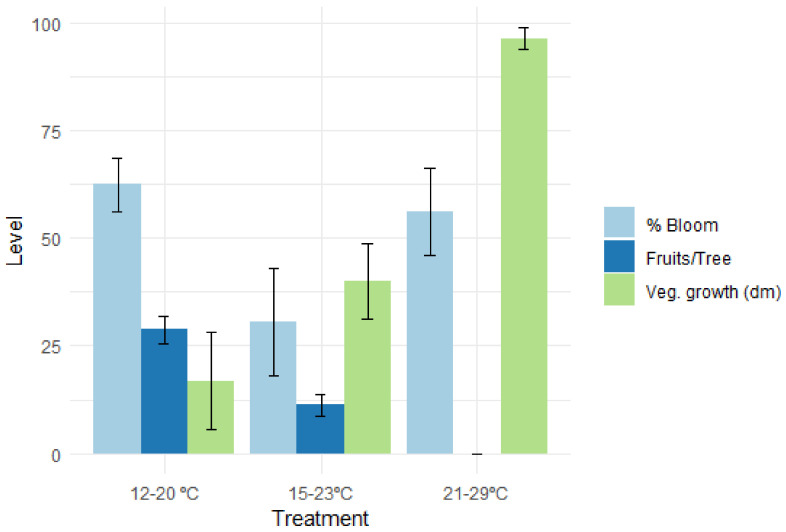
Vegetative and reproductive development in *Earligrande* peach under various night–day temperatures prior to bud break (temperature regimes lasted 3 weeks after chilling had been fully satisfied; daylength = 11 h) [32].

The sensitivity to high temperatures prior to the bloom has another aspect, which is demonstrated in peaches by comparing their vegetative and fruit set response under various temperature regimes (Figure 8). Clearly, under high temperatures, vegetative development is enhanced and the level of the fruit set is reduced [31]. This could be the result of competition between the two bud types or because of a specific negative effect of high temperature on normal flower development.

## 6. Conclusions

The awareness of the global warming phenomenon and its effect on chilling requirements for all *Prunus* species leads to an increased interest in the means to cope with it under orchard conditions. Breeding low-chilling-requiring cultivars that will also resist heat spells in winter is the most effective strategy but it takes a long time to achieve. We do have other means to fight global warming. The first factor that needs to be evaluated is the actual understanding of what chilling is and how we measure and record it under field conditions. The second is what are the means available for use when winter chilling accumulation is reduced. In this work, the dynamic model has been proposed as the best model now available for evaluating chilling accumulation and a few means are suggested to either reduce chilling requirements, or to compensate for insufficient chilling. In addition, it is stated that apart from the negative effects of high winter temperatures on chilling accumulation during dormancy, specific detrimental effects on floral buds of *Prunus* species can be observed due to winter heat spells.

## Figures and Tables

**Table 1:** The effect of dormancy-breaking agents on bud break in *Maravilla* peach. (Applied December 2001, Oil = 5%, DNOC = 0.12%, TDZ = thidiazuron. Figures in columns accompanied with different letter are significantly different by Duncan MRT at 5% level. Chilling accumulated, by December 7, only 4 chilling portions).

Treatment	Bloom Level (1–10)	Leafing Level (1–10)	Total Harvest Until April 22 (Kg/Ha)
Control	0.13 b	0 c	500 b
Oil–DNOC	1.00 b	0 c	1130 b
Oil–DNOC + Dormex 0.5%	2.25 b	0.6 c	3510 b
Oil + TDZ, 75 ppm	4.88 a	3.6 b	13,910 a
Oil + TDZ, 100 ppm	5.63 a	4.8 a	14,540 a

## Data Availability

Not applicable.
